# Impact of Microbial Protease Enzyme and Dietary Crude Protein Levels on Growth and Nutrients Digestibility in Broilers over 15–28 Days

**DOI:** 10.3390/ani11092499

**Published:** 2021-08-25

**Authors:** Abdul Jabbar, Muhammad Tahir, Ibrahim A. Alhidary, Mutassim A. Abdelrahman, Hani Albadani, Rifat Ullah Khan, Maria Selvaggi, Vito Laudadio, Vincenzo Tufarelli

**Affiliations:** 1 Department of Animal Nutrition, Faculty of Animal Husbandry and Veterinary Sciences, The University of Agriculture, Peshawar 25000, Pakistan; abduljabbar@upr.edu.pk (A.J.); tahir065@yahoo.co.uk (M.T.); 2 Faculty of Veterinary and Animal Sciences, University of Poonch, Rawalakot 12350, Pakistan; 3 Department of Animal Production, College of Food and Agriculture Sciences, King Saud University, Riyadh 11414, Saudi Arabia; ialahidary@ksu.edu.sa (I.A.A.); amutassim@ksu.edu.sa (M.A.A.); halbadani@ksu.edu.sa (H.A.); rukhan@aup.edu.pk (R.U.K.); 4 Faculty of Animal Husbandry and Veterinary Sciences, College of Veterinary Sciences, The University of Agriculture, Peshawar 25000, Pakistan; 5 Department of Agricultural and Environmental Science, University of Bari “Aldo Moro”, 70125 Bari, Italy; maria.selvaggi@uniba.it; 6 Department of DETO, Section of Veterinary Science and Animal Production, University of Bari “Aldo Moro”, 70010 Bari, Italy; vincenzo.tufarelli@uniba.it

**Keywords:** broiler, protease, dietary protein, growth, nutrient utilization

## Abstract

**Simple Summary:**

Protein is considered as the most expensive nutrient in animal diet and requires exogenous proteolytic enzymes for proper digestion in all living organisms. Thus, the present study evaluated different crude protein levels (CP) with or without exogenous protease in broilers diet over 15–28 days on their growth and nutrient digestibility. Based on the obtained findings, it is recommended a reduction in protein contents of diets from 21 to 19% when the protease enzyme is supplemented, to produce a beneficial impact on the growth and digestibility of broilers and also to reduce feeding costs.

**Abstract:**

In this trial, a 3 × 2 factorial design with different dietary crude protein levels (CP, 17, 19 and 21%) and two levels of exogenous protease (0 and 30,000 IU/kg) was used. A total of 540 two-week old broilers (Ross-308) was randomly allocated to experimental diets over 15–28 days of age. The interaction between dietary protein levels and enzyme supplementation showed that body weight gain was significantly (*p* < 0.05) higher in birds fed CP-19 (1114.7 g) and CP-21 (1108.8 g) with enzymes supplementation. Feed intake was higher (*p* < 0.05) in broilers fed with CP-17 than CP-19 with supplementation of the protease enzyme. Results also revealed that the feed conversion ratio (FCR) was significantly (*p* < 0.05) improved in birds fed with CP-19 and CP-21 and protease supplementation. Total tract N retention was lower (*p* < 0.05) in birds fed CP-17 with no enzyme than the other dietary groups. Similarly, the gross energy (GE) was significantly (*p* < 0.05) lower in birds fed CP-17 with or without the protease enzyme. Abdominal fat was higher (*p* < 0.05) in CP-17 (0.96%) without the protease enzyme. It was concluded that a diet at 19% CP with the protease enzyme improved the performance and nutrient digestibility in broilers over 15–28 days.

## 1. Introduction

The inclusion of exogenous protease may be an effective nutritional tool to improve growth and increase digestibility of crude protein and metabolizable energy in broiler diet [1]. Endogenous enzymes are secreted in animal body, but their concentration is not always sufficient for efficient protein digestion [2], especially during the growing phase, where feed intake substantially increases. Past experiments indicated that most of the crude protein (CP) went undigested without being completely utilized [3,4]. This undigested protein may be better utilized in the presence of the exogenous protease enzyme. Therefore, it is speculated that exogenous supplementation of enzymes may optimize the CP digestibility and growth performance of broiler chickens [5,6]. Previous studies have also demonstrated that nutrient density is more important for optimum growth and feed efficiency in broilers during the finisher phase than the starter phase [7,8,9].

To minimize the feed cost, a low-protein diet is recommended in broilers [10]; however, there is likelihood of less chance to obtain sufficient amount of essential amino acids, which may also compromise the conversion of non-essential amino acids [2]. Several exogenous enzymes are commercially available and being used in SBM-based diets to improve the growth of broiler chickens. Exogenous enzymes improve not only feed efficiency, but also reduce proteolytic fermentation, nutrients excretion in feces and bacterial toxins [11]. Moreover, the protease enzyme cleaves anti-nutritional factors, such as trypsin inhibitors, thus resulting in enhanced utilization of amino acids [2]. Earlier studies have also demonstrated that protease supplementation increased ileal digestibility, resulting in improved growth performance in broilers fed on low-protein diets [12]. In addition, little attention has been given to the digestibility of energy of nutrients in protease-supplemented birds. Many studies have documented inconclusive findings in broilers fed with different levels of CP [9,11,12]. Law et al. [12] concluded that digestibility of nutrients was higher in broilers fed with 19% CP than 16% co-supplemented with the protease enzyme during the finishing phase. Jabbar et al. [13] reported best results in term of performance and digestibility of nutrients in response to protease enzyme supplementation in broilers fed with 21% CP. Another study reported that exogenous protease supplementation in low energy-protein diets is helpful in achieving enhanced growth performance and lower abdominal fat in broilers in heat stress conditions [9]. In the published literature, discrepancies exist on the level of CP in broilers diet. We hypothesized that addition of the protease enzyme could reduce the level of CP with no compromise on the growth performance of broiler. Therefore, the objective of the present study is to evaluate different levels of CP with and without exogenous protease in broiler diet over 15–28 days on their growth and nutrient digestibility.

## 2. Materials and Methods

### 2.1. Ethical Approval

The study was approved by the Committee on Ethical and Animal Welfare, Faculty of Animal Husbandry and Veterinary Sciences, University of Agriculture, Peshawar.

### 2.2. Experimental Procedures

Day-old chicks were reared under standard protocol of feeding (CP 22%, 2700 Kcal/kg), environment temperature (35 °C in initial week and gradual decrease in the following week) and lighting (23:1 light and dark cycle). Wood shavings were used as bedding material with ad libitum access to drinking water. Using a 3 × 2 factorial design having three dietary protein levels (CP, 17,19 and 21%) and two levels of the protease enzyme (0 and 30,000 IU/kg), a total of 540 day-old male broiler chicks (Ross-308) were randomly allotted to 36 floor pens (*n* = 15 chicks/pen) from 15 to 28 days of age. There were six replicate pens per crude protein-enzyme supplementation subgroups. All the diets (pellet form) were isocaloric, having digestible amino acids level that met or exceeded the requirements [14]. The stocking density was 10 birds/m^2^. The house temperature was maintained at 25 ± 2 °C and humidity was 61%. Feed supply and consumption were weighed during the entire period. Weight gain in broilers was recorded on a weekly basis for each group at the end of experimental period. The diet was mixed with the exogenous protease enzyme (Pro Plus, Medixacell, Lahore, Pakistan) at two dose levels (0 and 30,000 IU/kg). Protease was derived from *Bacillus subtilis* and capable to break down protein and anti-nutritional factors in plants with the strength activity of 600,000 PROT units/kg. The protease activity of this enzyme was measured in PROT units, which was defined as the concentration of enzyme that releases 1 µmol of ρ-nitroaniline from 1 µM of substrate/min at 37 °C and pH of 9. Three diets having 17, 19 and 21% CP with same level of metabolizable energy were prepared as reported in Table 1.

### 2.3. Total Tract Digestibility Assay

Total tract N retention was determined on day 28 of the experiment using acid-insoluble ash as digestibility marker. On day 25 of the experiment, the marker was mixed in the experimental rations and fed to the birds (five birds per replicate), separated in metallic cages, till the end of the trial. On day 28 of the experiment, excreta samples were collected twice a day in plastic bags and stored at −20°C. The grinded feed and excreta samples were passed through a 0.5 mm sieve and stored at −20°C. Nitrogen (N) was determined through the Kjeldahl technique and multiplied by 6.25 to find out total tract N retention. Gross energy (GE) was determined using a bomb calorimeter [13]. On day 28, five birds per replicate were randomly selected to determine abdominal fat. Birds were sacrificed (cut the neck with sharp knife) and defeathered and abdominal fat was removed from around the abdominal organs and belly. Fat pads were weighed as percentage of live body weight.

### 2.4. Statistical Analysis

Data were analyzed by analysis of variance (ANOVA) using the general linear model (GLM) procedure of the statistical analysis system of SAS [15] to identify the main effects of diets, enzyme and their interaction by factorial design. When the interaction was found to be significant, Tukey’s test was applied to separate the means. A *p*-value less than 0.05 was found to be statistically significant.

## 3. Results

Data regarding the effects of different dietary protein and protease on broiler chick’s performance are presented in Table 2. The results show that body weight gain was significantly (*p* < 0.05) lower in the group fed CP-17, with no significant (*p* > 0.05) difference in feed intake. As a consequence, FCR was significantly (*p* < 0.05) higher in birds fed with CP-17. Similarly, body weight gain was significantly (*p* < 0.05) higher in birds treated with enzymes, while FCR was significantly (*p* < 0.05) lower in the same group. The interaction between protein levels and enzyme supplementation showed that body weight gain was significantly (*p* < 0.05) higher in birds fed with CP-19 and CP-21 with enzymes supplementation. Feed intake was significantly (*p* < 0.05) higher in birds fed with CP-17 than CP-19 with supplementation of protease enzyme. The results also revealed that FCR was significantly (*p* < 0.05) lower in birds with protease supplementation.

The effects of the different levels of protein and enzyme supplementation on digestibility indices and abdominal fat are given in Table 3. Results revealed that total tract N retention was significantly (*p* < 0.05) higher in birds fed with CP-19 and CP-21. The GE was significantly (*p* < 0.05) higher in the diet with CP-19 and CP-21 than CP-17. Abdominal fat was significantly (*p* < 0.05) lower in CP-19 and CP-21 than CP-17. Protease supplementation significantly (*p* < 0.05) improved total tract N retention and GE; however, abdominal fat (0.62%) was reduced (*p* < 0.05) in broiler chickens. The interaction showed that CP digestibility was lower (*p* < 0.05) in birds fed with CP-17 with no enzyme supplementation than the rest of the groups. Similarly, GE was significantly (*p* < 0.05) higher in birds fed with CP-19 and 21 plus protease supplementation than CP-17 without protease enzyme. Further, abdominal fat was significantly (*p* < 0.05) higher in CP-17 without protease enzyme than the other treatments.

## 4. Discussion

Enzyme supplementation in broiler diet is well known for its economic, environmental and nutritional advantages. In the present study, the body weight of birds was significantly higher when fed with CP-19 plus protease enzyme, while FCR and abdominal fat were significantly lower in the same group. In contrast, the growth performance, feed intake and feed efficiency were severely affected in birds fed with CP-17 with or without enzymes. This outcome was expected, since the lower CP accumulated body fat and resulted in decreased feed efficiency and utilization. Our findings are partially similar to the previous studies reporting that low CP diets negatively affect the performance and feed efficiency of broilers [2,16]. In the present study, it was obvious that CP-19 with enzyme supplementation was superior in terms of higher growth performance in than CP-21 without enzyme supplementation. There is also an economic aspect to these findings, since the broiler producers would opt to use CP-19 with protease supplementation to reduce feed-cost. Previous studies have attempted to decrease the CP content of broiler and ducks, which resulted in some noxious effects [17,18]. In the present study, total tract N retention and GE were significantly improved in birds fed with CP-19 with supplementation of protease. Previously, it has been reported that protease enzyme supplementation improved CP digestibility in broiler chickens [3,11,19,20]. Freitas et al. [21] reported that digestibility of protein is higher in high-CP than low-CP diets, in response to the exogenous protease enzyme. Although this behavior of the protease enzyme has resulted in inconsistent growth performance in some of the experiments in broilers [22], this was not the case in our study. In this experiment, the improvement in the digestibility indices resulted in a parallel increase in the growth performance of broilers. Furthermore, in the current study, GE was the highest in birds fed a diet with CP-19 and CP-21 and protease enzyme. Freitas et al. [21] reported improved digestibility of CP in broilers in response to low-CP in the diet. Similar to our findings, Vieira et al. [23] reported improved CP digestibility in protease-supplemented broilers, when compared with a low-CP diet. Moreover, Favero et al. [24] also reported a 5% improved digestibility of energy in broilers in the highest level of CP with protease supplementation. In contrast to our results, the metabolizable energy was higher in birds given a low-CP diet [3].

In the current study, the highest fat percentage was found in broilers fed with CP-17 with no protease enzyme supplementation. It is clear from this study that low protein diet promotes abdominal fat deposition. Abdominal fat of the carcass is an unfavorable trait and its presence decreases the consumers’ acceptability. It is one of the major drawbacks of the low-CP diet. In an experiment, fat content was reduced in broiler fed with low-CP diet [9]. Previous studies have reported a significantly higher percentage of abdominal fat in broiler at the end of experimental period fed with low protein diet [17,20], which has been attributed to higher calorie: protein ratio. It is inferred that exogenous protease helps to digest fats, thus increasing the availability of metabolizable energy, as recorded in the current study and reported previously [5]. Thus, it seems that excess energy beyond protein deposition is mainly accumulated as abdominal fat. In the current study, it was also clear that there was a slight difference in growth and digestibility in CP-19 and 21, elucidating that birds performed well under these two levels of CP when protease was supplemented in the diets. Previous studies have recommended a slight reduction in protein contents of the diets when the protease enzyme was supplemented to produce a beneficial impact on the growth and digestibility of broilers [9,13]. Further, it is more economical for farmers to use CP-19 than CP-21.

Therefore, based on the present findings, it was concluded that fed a diet at 19% CP with the protease enzyme improved the growth performance and nutrients digestibility during a trial period of 15–28 days.

## Figures and Tables

**Table 1:** Ingredients and chemical composition of diets over 15–28 days.

Ingredients (%)	Diet 17% CP	Diet 19% CP	Diet 21% CP
Corn	62.45	59.62	57.18
Soybean meal (44% CP)	9.27	11.08	14.51
Canola meal	7.00	7.00	8.00
Sunflower meal	5.71	6.00	5.00
Gluten meal (60% CP)	4.50	2.50	3.00
Poultry by-products	2.00	3.00	3.00
Gluten meal (40% CP)	1.21	2.60	2.81
Vegetable oil	1.00	2.19	2.04
Bone meal	1.00	1.00	1.00
Dicalcium Phosphate	1.00	1.00	0.65
Limestone	1.00	0.86	1.02
Salt	0.45	0.45	0.45
VitaVit-minerals premix ^1^	0.12	0.12	0.12
L-Lys HCl	0.55	0.42	0.23
DL-Met	0.28	0.20	0.14
Valine	0.99	0.71	0.46
Threonine	0.72	0.66	0.34
Isoleucine	0.43	0.36	0.00
Arginine	0.32	0.22	0.00
*Chemical composition*			
ME, kcal/kg	2950	2930	2920
Crude protein, %	17.00	19.00	21.00
Ca, %	1.00	1.00	1.00
Available P, %	0.45	0.45	0.45
Met + Cys, %	0.90	0.90	0.90
Methionine, %	0.59	0.56	0.53
Lysine, %	1.15	1.15	1.15
Valine, %	0.96	0.68	0.99
Threonine, %	0.83	0.72	0.89
Isoleucine, %	0.93	0.85	0.88
Arginine, %	1.19	1.09	1.21

^1^ Vitamin–mineral premix/kg contains the following per kg: vitamin A, 2,400,000 IU; vitamin D, 1,000,000 IU; vitamin E, 16,000 IU; vitamin K, 800 mg; vitamin B1, 600 mg; vitamin B_2_, 1600 mg; vitamin B_6_, 1000 mg; vitamin B_12_, 6 mg; niacin, 8000 mg; folic acid, 400 mg; pantothenic acid, 3000 mg; biotin 40 mg; antioxidant, 3000 mg; cobalt, 80 mg; copper, 2000 mg; iodine, 400; iron, 1200 mg; manganese, 18,000 mg; selenium, 60 mg; zinc, 14,000 mg.

**Table 2:** Body weight gain, feed intake and feed conversion ratio (FCR) of broilers fed different levels of crude protein and protease enzyme over 15–28 days.

Item	Body Weight Gain (g)	Feed Intake (g)	FCR (g/g)
CP ^1^			
CP-17	938.0 ^b^	1895.0	2.02 ^a^
CP-19	1098.4 ^a^	1857.1	1.69 ^b^
CP-21	1096.0 ^a^	1873.2	1.71 ^b^
Pooled SEM	76.33	102.45	0.11
Enzyme ^2^			
Without enzyme	1021.0 ^b^	1867.2	1.84 ^a^
CP + enzyme	1067.0 ^a^	1883.1	1.77 ^b^
Pooled SEM	45.66	124.00	0.12
CP × Enzyme			
CP-17_0_	899.0 ^d^	1875.0 ^ab^	2.08 ^a^
CP-17_E_	977.3 ^c^	1915.4 ^a^	1.96 ^b^
CP-19_0_	1082.2 ^b^	1858.0 ^ab^	1.71 ^c^
CP-19_E_	1115.0 ^a^	1856.4 ^b^	1.66 ^d^
CP-21_0_	1082.4 ^b^	1869.0 ^ab^	1.72 ^c^
CP-21_E_	1109.00 ^ab^	1877.4 ^ab^	1.69 ^cd^
Pooled SEM	78.33	106.55	0.11
*p*-Value			
CP	<0.001	0.1799	<0.001
Enzyme	0.0001	0.3301	0.0001
CP × Enzyme	0.0456	0.0383	0.0238

Means in the same column with different superscripts differ significantly (*p* < 0.05). ^1^ Diets containing different levels of CP: CP-1 = 17% CP; CP-2 = 19% CP; CP-3 = 21% CP (all these diets were with or without protease enzyme). ^2^ Protease enzyme at 30,000 UI/kg.

**Table 3:** Total tract N retention, gross energy (GE) and abdominal fat of broilers fed different levels of crude protein and protease enzyme over 15–28 days.

Item	Total Tract N Retention (%)	GE (Kcal/kg)	Abdominal Fat (%)
CP ^1^			
CP-17	66.00 ^b^	2889.0 ^b^	0.87 ^a^
CP-19	68.00 ^a^	2928.0 ^a^	0.59 ^b^
CP-21	68.25 ^a^	2930.0 ^a^	0.55 ^c^
Pooled SEM	1.74	103.55	0.01
Enzyme ^2^			
Without enzyme	66.08 ^b^	2904.0	0.72 ^a^
CP + enzyme	68.29 ^a^	2927.1	0.62 ^b^
Pooled SEM	2.42	203.45	0.01
CP × Enzyme			
CP-17_0_	62.71 ^d^	2876.0 ^b^	0.96 ^a^
CP-17_E_	64.00 ^c^	2902.0 ^ab^	0.77 ^b^
CP-19_0_	67.00 ^b^	2919.0 ^ab^	0.63 ^c^
CP-19_E_	68.19 ^ab^	2936.5 ^a^	0.55 ^d^
CP-21_0_	68.18 ^ab^	2917.0 ^ab^	0.57 ^d^
CP-21_E_	68.36 ^a^	2943.2 ^a^	0.53 ^d^
Pooled SEM	3.33	107.00	0.01
*p*-value			
CP	<0.001	<0.001	0.0348
Enzyme	0.0007	<0.001	0.0919
CP × Enzyme	0.0339	0.0009	0.0052

Means in the same column with different superscripts differ significantly (*p* < 0.05). ^1^ Diets contained different levels of CP: CP-1 = 17% CP; CP-2 = 19% CP; CP-3 = 21% CP (all these diets were with or without protease enzyme). ^2^ Protease enzyme at 30,000 UI/kg.

## References

[1] Hu J., Ingale S., Rathi P., Zhang J.Y., Kim I.H. (2020). Influence of exogenous acid protease in broiler chickens fed corn–soybean meal-based diets. J. Anim. Physiol. Anim. Nutr..

[2] Ndazigaruye G., Kim D.H., Kang C.W., Kang K.R., Joo Y.J., Lee S.R., Lee K.W. (2019). Effects of low-protein diets and exogenous protease on growth performance, carcass traits, intestinal morphology, cecal volatile fatty acids and serum parameters in broilers. Animals.

[3] Angel C.R., Saylor W., Vieira S.L., Ward N. (2011). Effects of a mono-component protease on performance and protein utilization in 7-to 22-day-old broiler chickens. Poult. Sci..

[4] Ding X.M., Li D.D., Li Z.R., Wang J.P., Zeng Q.F., Bai S.P., Su Z.W., Zhang K.Y. (2016). Effects of dietary crude protein levels and exogenous protease on performance, nutrient digestibility, trypsin activity and intestinal morphology in broilers. Livest. Sci..

[5] Cowieson A.J., Roos F.F. (2016). Toward optimal value creation through the application of exogenous mono-component protease in the diets of non-ruminants. Anim. Feed Sci. Technol..

[6] Saleh A.A., Dawood M.M., Badawi N.A., Ebeid T.A., Amber K.A., Azzam M.M. (2020). Effect of supplemental serine-protease from *Bacillus licheniformis* on growth performance and physiological change of broiler chickens. J. Appl. Anim. Res..

[7] Kamran Z., Sarwar M., Nisa M., Nadeem M., Mahmood S., Babar M., Ahmed S. (2008). Effect of low-protein diets having constant energy-to-protein ratio on performance and carcass characteristics of broiler chickens from one to thirty-five days of age. Poult. Sci..

[8] Cowieson A., Zaefarian F., Knap I., Ravindran V.R. (2017). Interactive effects of dietary protein concentration, a mono-component exogenous protease and ascorbic acid on broiler performance, nutritional status and gut health. Anim. Prod. Sci..

[9] Law F.L., Zulkifli I., Soleimani A.F., Liang J.B., Awad E.A. (2019). Effects of protease supplementation of low protein and/or energy diets on growth performance and blood parameters in broiler chickens under heat stress condition. Ital. J. Anim. Sci..

[10] Polat I., Yurtseven S., Sarica S. (2020). Supplementing diets for broilers that are low in crude protein and amino acids with protease. S. Afr. J. Anim. Sci..

[11] Mahmood T., Mirza M.A., Nawaz H., Shahid M. (2017). Effect of different exogenous proteases on growth performance, nutrient digestibility, and carcass response in broiler chickens fed poultry by-product meal-based diets. Livest. Sci..

[12] Law L., Zulkifli I., Soleimani F.A., Hossain M., Liang J. (2015). Nutrient digestibility of broiler chickens fed on a low-protein diet supplemented with mono-component proteases. Eur. Poult. Sci..

[13] Jabbar A., Tahir M., Khan R.U., Ahmad N. (2021). Interactive effect of exogenous protease enzyme and dietary crude protein levels on growth and digestibility indices in broiler chickens during the starter phase. Trop. Anim. Health Prod..

[14] Aviagen W. Ross 308: Broiler Nutrition Specification. https://tmea.aviagen.com/assets/Tech_Center/Ross_Broiler/RossBroilerNutritionSpecs2019-EN.pdf.

[15] SAS Institute Inc (2002). SAS/STAT User’s Guide.

[16] Yang H., Yang Z., Wang Z., Wang W., Huang K., Fan W., Jia T. (2015). Effects of early dietary energy and protein dilution on growth performance, nutrient utilization and internal organs of broilers. Ital. J. Anim. Sci..

[17] Namroud N.F., Shivazad M., Zaghari M. (2008). Effects of fortifying low crude protein diet with crystalline amino acids on performance, blood ammonia level, and excreta characteristics of broiler chicks. Poult. Sci..

[18] Wang Q.D., Zhang K.Y., Zhang Y., Bai S.P., Ding X.M., Wang J.P., Peng H.W., Tian G., Xuan Y., Su Z.W. (2020). Effects of dietary protein levels and protease supplementation on growth performance, carcass traits, meat quality, and standardized ileal digestibility of amino acid in Pekin ducks fed a complex diet. Poult. Sci..

[19] Ghazi S., Rooke J.A., Galbraith H., Bedford M.R. (2002). The potential for the improvement of the nutritive value of soya-bean meal by different proteases in broiler chicks and broiler cockerels. Br. Poult. Sci..

[20] Law F.L., Zulkifli I., Soleimani A.F., Liang J.B., Awad E.A. (2018). The effects of low-protein diets and protease supplementation on broiler chickens in a hot and humid tropical environment. Asian-Australas. J. Anim. Sci..

[21] Freitas D.M., Vieira S.L., Angel C.R., Favero A., Maiorka A. (2011). Performance and nutrient utilization of broilers fed diets supplemented with a novel mono-component protease. J. Appl. Poult. Res..

[22] Walk C.L., Pirgozliev V., Juntunen K., Paloheimo M., Ledoux D.R. (2018). Evaluation of novel protease enzymes on growth performance and apparent ileal digestibility of amino acids in poultry: Enzyme screening. Poult. Sci..

[23] Vieira S.L., Angel C.R., Miranda D.J.A., Favero A., Cruz R.F.A., Sorbara J.O.B. (2013). Effects of a monocomponent protease on performance and protein utilization in 1-to 26-day-of-age turkey poults. J. Appl. Poult. Res..

[24] Favero A., Maiorka A., Rocha C., Appelt M.D., Sorbara J.O.B. (2009). Effect of protease enzyme on performance and ileal digestibility of broilers grown to 42 days of age in floor pens. Int. Poult. Sci. Forum Atlanta Ga. Abstr. Pap. M28.

